# Fabrication of bioactive nanocomposites from chitosan, cress mucilage, and selenium nanoparticles with powerful antibacterial and anticancerous actions

**DOI:** 10.3389/fmicb.2023.1210780

**Published:** 2023-07-21

**Authors:** Mohsen M. El-Sherbiny, Mohamed I. Orif, Mohamed E. El-Hefnawy, Sultan Alhayyani, Soha T. Al-Goul, Rawan S. Elekhtiar, Hoda Mahrous, Ahmed A. Tayel

**Affiliations:** ^1^Department of Marine Biology, Faculty of Marine Sciences, King Abdulaziz University, Jeddah, Saudi Arabia; ^2^Department of Marine Chemistry, Faculty of Marine Sciences, King Abdulaziz University, Jeddah, Saudi Arabia; ^3^Department of Chemistry, College of Sciences and Arts, King Abdulaziz University, Rabigh, Saudi Arabia; ^4^Department of Fish Processing and Biotechnology, Faculty of Aquatic and Fisheries Sciences, Kafrelsheikh University, Kafr el-Sheikh, Egypt; ^5^Genetic Engineering and Biotechnology Research Institute, University of Sadat City, Sadat, Egypt

**Keywords:** anticancer, antimicrobial, biocidal activities, biopolymers nanocomposites, biosynthesis

## Abstract

Natural bioactive alternatives are the utmost requests from researchers to provide biosafe and effectual health-guarding agents. The biopolymers chitosan nanoparticles (NCT), mucilage of cress seed (GCm; *Lepidium sativum*), and GCm-mediated selenium nanoparticles (GCm/SeNPs) were innovatively employed for fabricating novel bioactive natural nanocomposites (NCs) with elevated bioactivities as bactericidal (against *Salmonella typhimurium* and *Staphylococcus aureus*) and anticancer (against CaCo-2 and HeLa cells). The SeNPs were successfully generated with GCm, and different NCs formulations were fabricated from NCT:GCm/SeNPs amalgam ratios including T1, T2, and T3 with 2:1, 1:1, and 1:2 ratios, respectively. The infrared analysis of synthesized molecules appointed apparent physical interactions among interacted molecules. The average particles’ sizes and charges of molecules/NCs were (12.7, 316.4, 252.8, and 127.3 nm) and (−6.9, +38.7, +26.2, and −25.8 mV) for SeNPs, T1, T2, and T3, respectively. The biocidal assessment of NCs indicated that T1 was the strongest antibacterial formulation, whereas T3 was the superior anticancer amalgam. These NCs formulations could exceed the biocidal potentialities of standard biocides. T1-NC could cause severe destructions/deformations in challenged *S. typhimurium* within 9 h, whereas T3-NCs induced apparent fluorescent apoptosis signs in treated HeLa cells. The prospective applications innovatively designed biocidal natural NCs that are recommended for controlling pathogenic bacteria and fighting cancerous cells.

## 1. Introduction

Biopolymers provided numerous valuable advantages for human health and nutrition, with their outstanding properties that include biodegradability, biocompatibility, highest biosafety, and biomolecules-carrying ability, in addition to biopolymers having bioactivities (e.g., antimicrobial, antioxidant, bio-chelating, anticancerous, and immune-stimulation properties) (Sivakanthan et al., 2020; Baranwal et al., 2022). The natural biopolymers and polysaccharides could be achieved from numerous sources, including plant materials, microorganisms, marine sources, and insects (Sun et al., 2022); they can be effectually transformed into nanoforms via different protocols to influentially augment their bioactivities and potentialities for environmental sectors, drugs/genes delivery, pharmaceutical applications, and food/nutrition disciplines (Singh et al., 2017).

Chitosan (Ch) biopolymer (the bioactive deacetylated form of chitin) was efficaciously extracted from diverse resources, e.g., crustacean wastes, fungal mycelia, insects’ skeletons, etc. (Kou et al., 2021). The chitosan nanoparticles (NCT) had increased attention for applications in drug encapsulation/delivery, pathogen control, agricultural fertilization, environmental protection, and health guarding (Jhaveri et al., 2021).

The nanocomposites (NC) are the conjugates from diverse material complexes; the biopolymer-based NCs should have one dimension on a nanometer scale (e.g., ≤ 1,000 nm; Shameem et al., 2021). The Ch and other hydrophilic biopolymer NCs have superb importance in nanobiotechnology for delivering therapeutic agents, genes, nutraceuticals, anticancerous agents, and antibiotics, as well as constructing engineered tissues with maximum biocompatibility, biodegradability, and nontoxicity (Kobayashi and Nakazato, 2020; Gu et al., 2021; Messias de Souza et al., 2022).

The plant-derived biomolecules, nanomaterials, and NCs are sustainable generous sources for most biotechnological approaches (Taban et al., 2021); their constituents from phytochemicals and bioactive compounds were always the amazing solution to generate novel effectual anticancer, antimicrobial, antiviral, and diseases control (Godlewska-Z Yłkiewicz et al., 2020). Garden cress (i.e., *Lepidium sativum*, belongs to Brassicaceae family), syn. Common name: Thufa or Hab el Rashaad” in Arab countries,” is an edible fast-growing herb, which disseminates in most tropical regions (Prajapati et al., 2014). The seeds of cress contain elevated ratios of proteins (~ 25%), carbohydrates (35%–55%), and lipids (15%–25%; Fahami and Fathi, 2018a). The mucilage of cress seed (GCm) is a biopolymer with galactomannan as the main composition and comprises diverse sugar types in its construction; GCm has an anionic nature and elevated nutritional and functional values including antioxidant and antimicrobial properties (Prajapati et al., 2014; Fahami and Fathi, 2018b). The existence of two uronic acids in GCm (glucuronic – galacturonic acids) provides it with poly-electrolyte properties (Karazhiyan et al., 2011). Moreover, the functional, physicochemical, nutritional, and bioactivity physiognomies of GCm suggested its potentiality as a promising pharmaceutical excipient, food supplement, and phyto-medicine (Taheri and Razavi, 2015; Othman et al., 2019).

The encapsulation/stabilization of different nanometals (e.g., silver and selenium) within the GCm matrix was recently achieved to provide a more synergistic antimicrobial activity to the innovative composites (Haj Bloukh et al., 2021; Ibrahim et al., 2021; Shehab et al., 2022).

The element selenium (Se) is a non-metallic, natural, and vital micronutrient for most mammals (at daily needs of 30–300 μg), which could be principally acquired from supplements and/or regular diets (Lenz and Lens, 2009). The zero state of Se oxidation (e.g., Se^0^) is regularly present in their nanoparticles (SeNPs) and has minimal toxicity and commendable bioavailability regarding the other Se oxidation states (Wang et al., 2007). The biogenic (green) synthesis of SeNPs has gained great prominence for applications in nutritional, medicinal, and pharmaceutical disciplines because they generate NPs with high stability, no-aggregations, maximum biosafety, and effectuality, compared to other syntheses approaches (physical–chemical; Wadhwani et al., 2016; Salem and Fouda, 2021; Spyridopoulou et al., 2021; Salem, 2023). The non-biogenic syntheses frequently involved low pH, high temperatures, elevated coasts, and hazardous chemicals (Baranwal et al., 2018; Salem, 2023), which increases the NPs toxicity and risk for human handling/consumption, whereas the biosynthesized SeNPs have non-toxic and eco-friendly natures (Ullah et al., 2022). Among the diverse nanometal types, SeNPs possess the selectivity as anticancer agents to attack cancerous cells, with no or minimal biotoxicity to somatic normal cells (Huang et al., 2013; Menon et al., 2018); the biogenic SeNPs also exhibited better bioavailability and greater chemopreventive bioactivity, with the lowest toxicity, compared with organic/inorganic Se compounds (Spyridopoulou et al., 2021; Vundela et al., 2022). Accordingly, the biogenic SeNPs and their conjugations were considered the most promising anticancerous NCs for treating different cancer types (Vahidi et al., 2020; Hashem and Salem, 2022; Ullah et al., 2022).

Although the SeNPs anticancer actions remain unclear, some presumed activities were suggested including the differential cytotoxicity action in cellular cancers, the pre-oxidative SeNPs transformation (due to acidic pH and redox perturbations of cancerous cells), disruption of cells’ mitochondrial membrane causing the increase/trigger of free radicals production, leakage of mitochondrial proteins stressing the endoplasmic reticulum, and the reduction of angiogenic signaling in cancerous cells inhibiting the growth/proliferation of cells and triggering cells’ apoptosis via the stimulation of caspases (Menon et al., 2018; Gunti et al., 2019; Ullah et al., 2022).

The biogenic SeNPs antibacterial activities were recurrently reported and confirmed, which involved the generation of reactive oxygen species “ROS”, penetration of inside cells, and interaction/suppression of vital cellular organelles, as well as obstructing metabolic pathways and disturbing cell membranes synthesis and permeability (Wadhwani et al., 2016; ElSaied et al., 2021; Gad et al., 2021).

The conjugations of biosynthesized SeNPs within coating biopolymers/polysaccharides (e.g., chitosan, cellulose, cress extract, curcumin, etc.) were evidenced to provide them with much biosafety and effectuality as powerful antimicrobial agents, with low biotoxicity potentials (Abu-Elghait et al., 2021; Alghuthaymi et al., 2021; Gad et al., 2021; Shehab et al., 2022).

Accordingly, we aimed to fabricate innovative NCs from NCT, GSm, and their mediated SeNPs to augment their bioactive potentialities as antibacterial and anticancer formulations from natural sources and with high efficiencies. The innovations of this study are the constructions of these novel biopolymer NCs, which could provide powerful bioactivities (antibacterial and anticancer) and biosafety compared to standard biocides.

## 2. Materials and methods

### 2.1. Materials and reagents

Garden cress (*L. sativum* L) seeds were attained from ARC “Agricultural Research Center, Giza, Egypt”. The entire used chemicals, dyes, media, and reagents were procured from Sigma-Aldrich Co. (St. Louis, MO, United States) unless other sources are mentioned.

### 2.2. GCm mucilage extraction

The extraction of GCm from *L. sativum* seeds was modified from Karazhiyan et al. (2011). The seeds were slightly crushed in a porcelain mortar and the materials were soaked in deionized water (DW) with a ratio of 1:30 (w/v) at 36°C and the pH adjusted to 10 ± 0.2 using 1 M NaOH. The mixture was finely stirred (115 × *g*) for 40 min, then the mucilage was detached from the seeds’ residues using a double cloth filter. Ethanol (96%) precipitation was employed by adding an equal volume of ethanol to the extract and keeping them at 4 ± 1°C for 130 min, then the precipitated GCm was separated through centrifugation (4,800 × *g*) and was freeze-dried.

### 2.3. Preparation of GCm/SeNPs

The reduction/mediation of SeNPs with GCm was adopted from the recently described method (Shehab et al., 2022). Sodium selenite solution (Na2SeO_3_, 10 mM) in DW was prepared, then 20 mL of it was amalgamated with an equal volume of GCm solution (0.1%, w/v) and stirred (655 × *g*) for 90 min. While stirring, 5 mL of aqueous ascorbic acid solution (28.4 mM) was gradually dropped into the mixture and stirring was sustained in the dark for an additional 60 min. The development of a brownish-orange color mixture indicated the GCm/SeNPs formation, which was harvested by centrifuging (SIGMA; 2–16 KL, Osterode am Harz, Germany; 10,700 × *g*, 25 min), and was washed with DW to eliminate excessive materials, re-centrifuged, and freeze-dried.

### 2.4. Construction of NCT/GCm/SeNPs nanocomposite

The following protocol was employed for GCm/NCT nanocomposite construction depending on the difference between the biopolymer charges:

Firstly, the GCm (or GCm/SeNPs complex) was dissolved in DW at 0.1%, w/v ratio, whereas Ch (CAS Number: 9012-76-4; molecular weight ~ 100 kDa; deacetylation ≥80%) was dissolved with the same ratio in diluted (1.5%, v/v) acetic acid. The solutions were sonicated for 15 min with “Branson Ultrasonics, Sonifier S250A, Danbury, CT” before conjugations. A measurement of 20 mL of GCm/SeNPs solution was amended with 200 mg of Na-tripolyphosphate (TPP), dissolved well, and dropped into NCT solution at 300 μL/min rate in three different trials; in trial (T1), GCm/SeNPs solution was dropped into 10 mL of NCT solution; in trial (T2), GCm/SeNPs solution was dropped into 20 mL of NCT solution; whereas in trial (T3), GCm/SeNPs solution was dropped into 40 mL of NCT solution. The NCT solutions were vigorously stirred (740 × *g*) throughout dropping and for 30 min after dropping. The formed NCs were collected with centrifugation (11,100 × *g*), washed with DW, re-centrifuged, and freeze-dried. To attain purer SeNPs, the DW washing was repeated five times followed by centrifugation to detach most of the GCm.

### 2.5. Characterization of nanomaterials/nanocomposites

#### 2.5.1. Fourier transform infrared “FTIR” spectroscopic analysis

The infrared (IR) spectroscopic spectra of employed compounds/composites in the investigation, e.g., NCT, GCm, GCm/SeNPs, NCT/GCm, and NCT/GCm/SeNPs, were assessed (FTIR; FT-IR-360, JASCO, Japan) to appraise the potential biochemical bonds/groups in used compounds and the interactions among them in the NCs. The compound/composite powders were firstly intermingled with KBr, and their IR transmission spectra were perceived within a wavenumber range of 4,000–450 cm^−1^.

#### 2.5.2. Assessment of particles’ size and charges (zeta-potential)

The synthesized nanomaterials/NCs were dissolved in DW, and sonicated for 5 min at 40 W, then their Ps, surfaces’ charges, and Ps distribution were assessed via the DLS “Dynamic light scattering” approach, employing Malvern^™^ Zetasizer (Malvern, Worcestershire, UK). The valuations were triplicated at 25 ± 1°C.

#### 2.5.3. Electron microscopy imaging

The scanning electron microscope “SEM, JEOL, IT100, Tokyo, Japan” and Transmission electron microscope “TEM, JEM-100CX, JEOL” were employed for screening the apparent shape, Ps, and distributions of nanomaterials. The NCs (T1, T2, and T3) suspensions were sonicated, mounted onto self-adhesive carbon discs, coated with palladium/gold (Polaron Inc., E5100 II, Hatfield, PA), and inspected using SEM at 10 kV operating acceleration. Besides, the plain SeNPs physiognomies were appraised with TEM after dispersion in DW, sonication, and drop-casting onto TEM-coated grids.

### 2.6. *In vitro* antibacterial assessment

The antibacterial powers of compounds/NCs (Ch, GCm, SeNPs, T1, T2, and T3 formulations) were qualitatively/quantitatively assessed against Gram-negative (G^−^) bacteria (*Salmonella enterica* serovar Typhimurium; *S. typhimurium* ATCC23852) and the Gram-positive (G^+^) strain (*Staphylococci aureus*; ATCC25923). Nutrient broth and agar (NB and NA) were operated for bacterial growth and challenged with aerobic conditions at 37 ± 1\u00B0C°. Ampicillin (Merck^™^; Darmstadt, Germany) was the employed standard antibiotic for comparing the activities, with identical challenging conditions.

#### 2.6.1. The qualitative assay

The ZOI “inhibition zone, disc diffusion assay” was applied for qualitative assessment of antibacterial activity. Bacterial cultures were spread (swapped) onto NA plates then sterile paper discs (impregnated in 0.1% aqueous solutions of compounds/nanocomposites) were positioned onto inoculated NA surfaces. The ZOIs appeared after plate incubation for 18 h–24 h were precisely measured (Tayel et al., 2011).

#### 2.6.2. The quantitative assays

The MIC “minimum inhibitory concentration, mg/L” were assessed from each compound/nanocomposite toward challenged bacterial strains (Tayel et al., 2011). In NB-contained tubes, serial dilutions from each agent were made to range from 1 to 100 μg/mL, then the tubes were inoculated with bacterial culture and incubated for 24 ± 2 h. After that, 100 μL from tubes that had no observable turbidity (no bacterial growth) were plated onto NA plates without the agents and incubated for a further day. The growth-free tubes and plates indicated the MIC values for each bacterium.

#### 2.6.3. SEM visualization of bactericidal action

The bacterial (*S. typhimurium*) deformation/distortions in cellular morphology were screened after exposure to (T1) of NCT/GCm/SeNPs using SEM and after exposure and incubation with NC (at 15 mg/L concentration) for 0 h, 3 h, 6 h, and 9 h. The logarithmic-grown bacteria were inoculated into NB that contained the NC, and after incubation, bacteria were harvested by centrifugation (4,800 × *g*) and fixed for 30 min with glutaraldehyde (2.5%), paraformaldehyde (2.0%), and Na-cacodylate buffer (0.1 M, pH 7.36). The cells were dehydrated using successive concentrations of ethanol, then dried using Auto-Samdri-815 critical-point drier (Tousimis, Rockville, MD) coated with palladium/gold, and inspected with SEM for the appearance of distortions/deformations in exposed cell’s morphology/structures (Elnagar et al., 2021).

### 2.7. Screening The anticancer bioactivity

#### 2.7.1. Cancerous cell lines

The CaCo-2 (adenocarcinoma) and HeLa (cervical carcinoma) cell lines were attained from ATCC “American Type Culture Collection, Rockville, United States”; the cells were cultured/enriched in RPMI-1640 “Roswell Park Memorial Institute medium” and supplemented with 10% FBS “fetal bovine serum” and antibiotic mixture (75 U/mL of penicillin and streptomycin). Cells were upheld as monolayers at 37°C in a 5% CO_2_ humidified atmosphere. Cisplatin was employed for comparing the anticancerous potentiality as the positive control.

#### 2.7.2. MTT cytotoxicity assessment

The cancerous cells were planted individually in 96-well, flat-bottom microtiter plates containing supplemented D-MEM media (~1 × 10^4^ cells/well) and incubated for 24 ± 1 h at the prior conditions. Successive concentrations (e.g., from 0 to 100.0 μg/mL) from compounds/NCs (Ch, GCm, SeNPs, T1, T2, and T3 formulations) were amended into cell-holding wells and then incubated (37°C, 5% CO_2_, 24 h). Consequently, MTT “3-(4,5-Dimethylthiazol-2-yl)-2,5-diphenyltetrazolium bromide” at 5.0 mg/mL concentration was appended to inoculated wells and incubated for a further 4 h. After medium detaching from wells, 100 μL of DMSO was appended to wells, mildly vortexed for 23 min, and their absorbance (at 570 nm) was colorimetrically measured, with regard to the prepared standard curve (Spyridopoulou et al., 2021).

#### 2.7.3. Appraisal of HeLa cells’ apoptosis via fluorescent staining

The potential apoptosis/necrosis signs in HeLa cells, after treatment with (T3) of NCT/GCm/SeNPs nanoconjugate, were screened via fluorescent staining and imaging using two assessment protocols. The first employed the combined fluorescent staining with AO/PI “acridine orange/propidium iodide” (Alalawy et al., 2020), where cancerous cells’ suspension (~200–250 cells/μL) was exposed to T3 at its IC_50_ concentration and incubated at 37 ± 1°C in 5% CO_2_ humidified air for 1 and 2 days. The exposed and untreated cells were then washed using PBS “Phosphate-buffered saline”, (pH = 7.4) and stained for 22 min with PI (4 mg/mL) and AO (10 mg/mL) in the dark.

In the second protocol (DAPI staining, 4,6-diamidino-2-phenylindole), the cells were challenged with the same conditions then stained with DAPI stain at 1 *μ*g/mL concentration, incubated for 1 h, and then photographed. Fluorescent images were taken via an Olympus microscope (BX51, Tokyo, Japan) to illustrate the potential apoptosis indicators.

### 2.8. Statistical analysis

The SPSS package “V 17.0, SPSS Inc., Chicago, IL” was used for statistical computing. Means of triplicates were computed, and the student t-test and ANOVA (one-way) were employed for assessing the data significance at *p* ≤ 0. 05.

## 3. Results and discussion

### 3.1. Infrared analysis of produced materials

**Figure 1:**
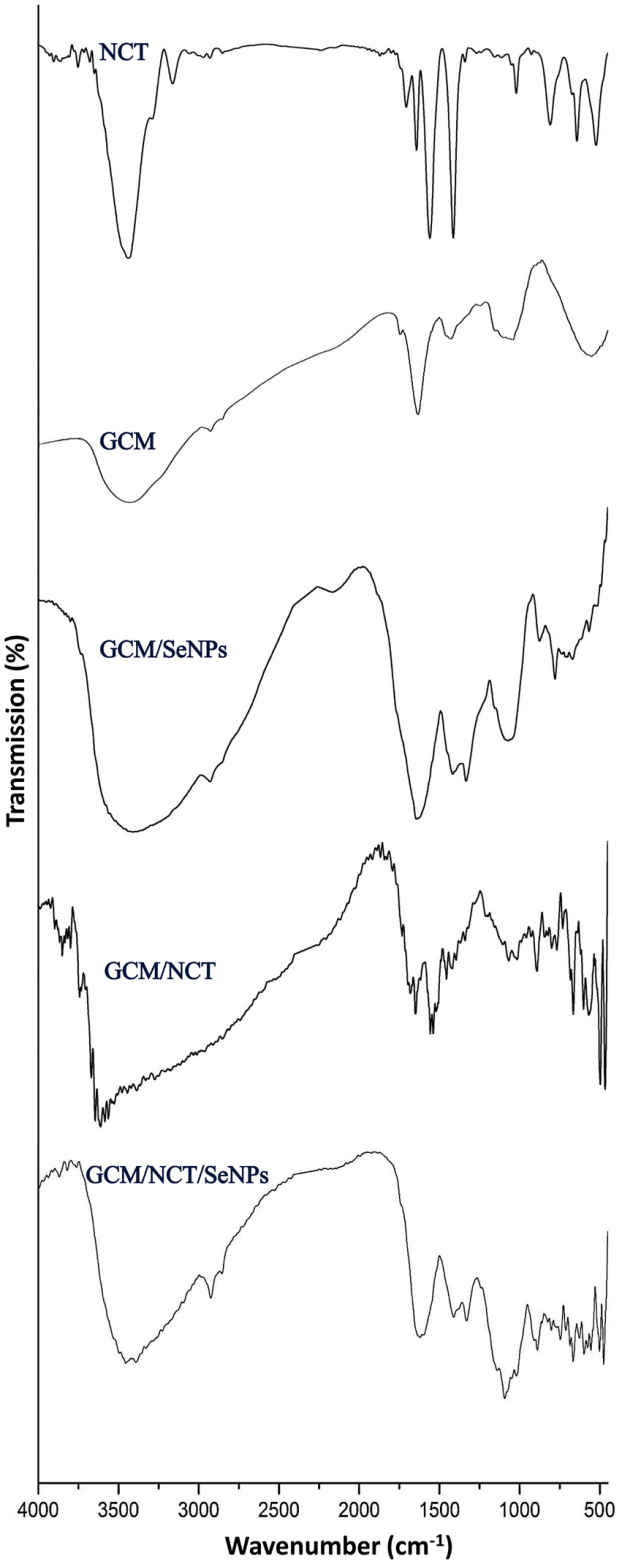
FTIR analysis of generated and composited materials including nanochitosan (NCT), garden cress mucilage (GSM), and selenium nanoparticles (SeNPs).

The extraction and conjugation of bioactive molecules (e.g., NCT, GCm, GCm/SeNPs, GCm/NCT, and NCT/GCm/SeNPs) were validated via the analysis of their FTIR spectra (Figure 1).

For the NCT, the FTIR spectrum appoints the fundamental bonds/groups that designate the bulk Ch (Figure 1-NCT). The key indicative peaks in the plain NCT spectrum included 3441.05 cm^−1^ (vibrated stretching of N − H and O − H), 2924.18 cm^−1^ (vibrated stretching of aliphatic C − H), 3073.27 cm^−1^ (vibrated stretching of CH_2_), 3027.81 cm^−1^ (vibrated stretching of C − H_3_); 1701.34 cm^−1^ (stretched C=O of amide I), 1666.74 cm^−1^ (vibrated stretching of NH in amide II), 1113.13 cm^−1^ (−OH vibrated stretching of C3), and 1037.88 cm^−1^ (−OH vibrated stretching of C6; Wazed et al., 2010; Alsharari et al., 2018). Furthermore, in the NCT spectrum, the 3441.05 cm^−1^ band, which habitually appears with lower intensity/wideness than bulk Ch, is an indicator of reduced −H bonding, which results from its interactions with cross-linked TPP (Tayel et al., 2020). The sharp peak that appears (1628.42 cm^−1^) is indicative of the linkage between TPP and the NH₄ of NCT (Alalawy et al., 2020).

The key biochemical bonds were presented in the GCm spectrum (Figure 1-GCM), which included the peaks at 1091.52 cm^−1^ (indicated weak stretching C–O), at 1422.47, and 1628.75 cm^−1^ (symmetric/asymmetric carboxylate groups). At 2926.42 cm^−1^, the sharp peak denoted the symmetric/asymmetric C–H stretching, whereas the bands within 3,000–2,800 cm^−1^ range signified the C–H vibrations (e.g., CH, CH_2_, and CH_3_ stretched and bent vibrations), which could occasionally overlap with O–H (Razmkhah et al., 2017; Fahami and Fathi, 2018a,b). The weak peak at 1149.14 cm^−1^ denoted the occurrence of monosaccharides (e.g., mannose and glucose; Moniri et al., 2020; Akl et al., 2021).

Numerous bands emerged after SeNPs synthesis and conjugation with GCm (Figure 1-GCM/SeNPs), e.g., in the range of 460–745 cm^−1^, at 772.62 cm^−1^, at 875 cm^−1^, at 1334 cm^−1^ and at 2181 cm^−1^, which indicates the formation of novel bonds between Se ions and GCm molecules (Shehab et al., 2022). Two bands disappeared in the GCm/SeNPs spectrum, compared with the GCm spectrum (e.g., at 1248 and 1752 cm^−1^), which indicates the breakage and occupation of these bonds after interaction with SeNPs (Shehab et al., 2022).

The NCT interactions with GCm were evidenced from their combined spectrum (Figure 1-GCM/NCT), where numerous beaks emerged after the biopolymers’ conjugation (e.g., in the ranges of 500–1,100 cm^−1^, 133–1700 cm^−1^, and 3,300–3,800 cm^−1^), which strongly indicated the formation of novel bonds between the two biopolymers. The combined molecules spectrum (Figure 1-GCM/NCT/SeNPs) emphasized the occurrence of different biochemical bonds from each involved compound, which may appoint more physical interactions rather than chemical interactions between the conjugated compounds (Fahami and Fathi, 2018b).

### 3.2. Structural physiognomies of synthesized nanomaterials/composites

**Figure 2:**
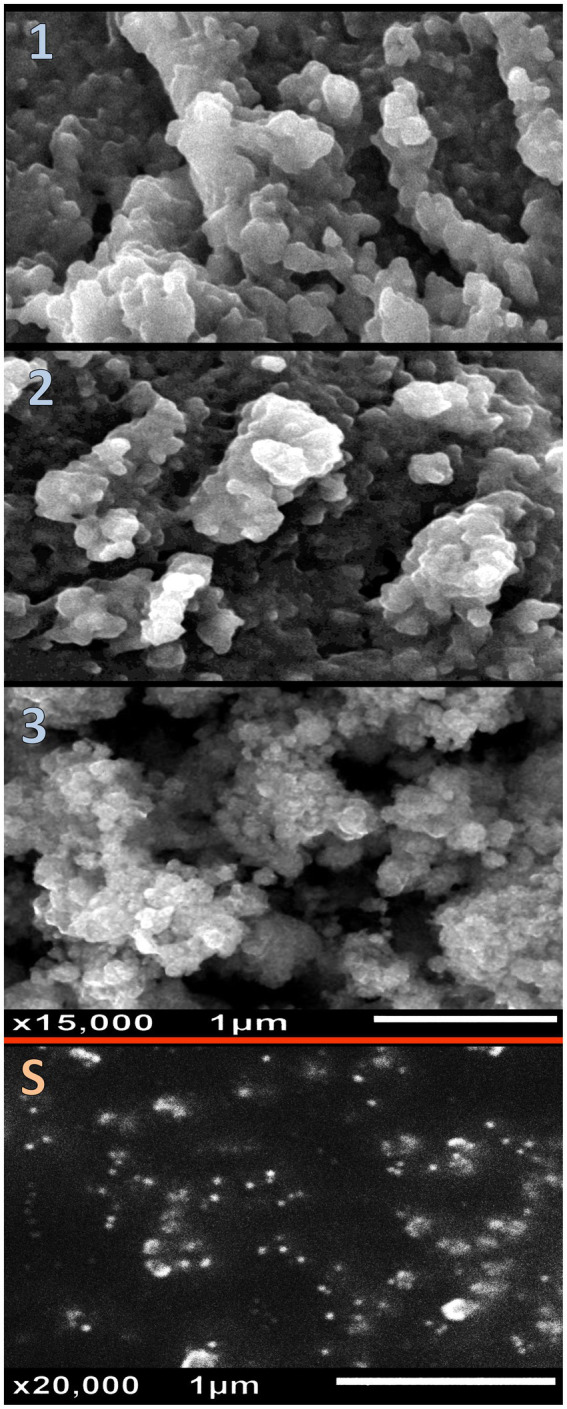
Structural physiognomies of synthesized nanomaterials/composites illustrating the nanocomposites of nanochitosan: garden cress mucilage ratios of 2:1 (1), 1:1 (2), and 1:2 (3), and pare selenium nanoparticles (S).

The structural physiognomies of synthesized nanomaterials/composites (e.g., Ch, GCm, SeNPs, and the different NCs of NCT:GCm/SeNPs) were appraised via DLS analysis (Table 1) and electron microscopy imaging (Figure 2). The bare Ch and GCm had a mean particle size (Ps) diameter of >1,000 nm and their surfaces were charged with +39.1 and –32.6 mV, respectively (Table 1). The bare SeNPs carried negative (−26.9 mV) charges and had mean Ps of 12.7 nm. The formulations from NCT:GCm at concentrations of 2:1, 1:1, and 1:2 (T1, T2, and T3, respectively) exhibited diverse Ps means and charges; the Ps mean became smaller with the decrease in NCT content in the formulations. Both T1 and T2 had positive charges (+38.7 and +26.2 mV) on their particles’ surfaces, whereas T3 particles were negatively (−25.8 mV) charged. The electron microscopy (SEM) imaging of formulated NCs (T1, T2, and T3) and bare SeNPs indicated their semi-spherical shapes, dispersion, and Ps distribution (Figure 2). The further characterization of GCm/SeNPs (e.g., via UV-visual assessment, XRD, and TEM) is additionally provided in the Supplementary materials (Supplementary Figures S1, S2, S3, respectively).

**Table 1:** Structural attributes of generated molecules/composites.

Trial code	NCT:GCm/SeNPs ratio	Mean particles’ diameter (nm)	Particles’ size range (nm)	Zeta potential (mV)	PDI
Ch	1:0	> 1,000	–	+39.1	0.679
GCm	0:1	> 1,000	–	˗32.6	0.824
SeNPs	–	12.7	4.6–34.1	˗26.9	0.345
T1	2:1	316.4	192.2–765.5	+38.7	0.447
T2	1:1	252.8	124.8–582.1	+26.2	0.533
T3	1:2	127.3	73.9–315.2	˗25.8	0.497

The attained Ps from the microscopic images matched the recorded Ps in the DLS analyses (Table 1). The SeNPs were well-dispersed with homogenous Ps and shapes. The T3 NCs (contained 1 NCT: 2 GCm/SeNPs) had the smallest Ps, compared to T1 and T2 NCs. Many aggregated particles were observed in the T2 and T3 NCs.

The effectual formation of NCs (T1, T2, and T3) validated the novel employed procedure for their synthesis and could encourage the application of this protocol for synthesizing more NCs from further biopolymers. The main reason that was suggested for achieving current NCs is the strong opposite charges on Ch and GCm, which was formerly reported for other NCs based on Ch conjugation with negatively charged biopolymers (e.g., alginate, carrageenan, ulvan, fucoidan, etc.) (Piras et al., 2015; Manivasagan and Oh, 2016; Venkatesan et al., 2016; Almutairi et al., 2021); the differences in surface charges between positive Ch and other negative polymers led to the formation of stable NCs with synergistic bioactivities (Manivasagan and Oh, 2016; Venkatesan et al., 2016). The cationic Ch and anionic GCm could combine to develop an exclusive polyelectrolyte complex (PLC) (Meng et al., 2010); the net charges of generated PLC depended on the ratios of involved electrolytes (Manivasagan and Oh, 2016). Zeta potential (e.g., surface charge) can significantly inspire NPs stability in suspension via electrostatic repulsion among particles, which is influential for the bioactivities of NCs (Larsson et al., 2012).

### 3.3. *In vitro* biocidal activities (antibacterial and anticancer) of examined compounds/NCs

The biocidal activities (antibacterial and anticancer) of examined compounds/NCs, compared to standard biocidal agents, are illustrated (Table 2). The antibacterial potentialities of entire agents/NCs were verified toward *S. typhimurium* (Gram-negative) and *S. aureus* (Gram-positive) strains; the NCs (T1, T2, and T3), respectively were significantly the most forceful and biocidal formulations against both strains. The most effectual antibacterial NCs was the T1, comparing with the MICs and ZOIs values of the other NCs. Examples of qualitative antibacterial activity images (inhibition zones) are provided in the Supplementary materials (Supplementary Figure S4). The antibacterial results of the T1 composite were comparable to ampicillin (standard bactericide) as no significant difference was calculated between their effects. The following powerful agents were the T2, T3, SeNPs, GCm, and Ch, respectively. *S. typhimurium* was generally more sensitive than *S. aureus* to the entire agents. The MIC values of NCs against *S. typhimurium* ranged from 15.0 mg/L (for T1) to 27.5 mg/L (for T3) compared to 47.5 mg/L for ampicillin. For *S. aureus*, the least MIC of NCs was 17.5 mg/L (T1) and the highest was 32.5 mg/L (T3), compared to 42.5 mg/L from ampicillin. The MICs of NCs were generally much lower (more effective) than their individual components (Ch, GCm, and SeNPs), which validates the antimicrobial synergism between these compounds. Both *S. typhimurium* and *S. aureus* were employed as models for G^−^ and G^+^ bacteria; other challenging bacterial strains could be suggested for more reliable results in prospective works. Interestingly, the entire NCs exhibited remarkable antimycotic (against *Candida albicans* ATCC-24433) and antifungal (against *Aspergillus flavus* ATCC-16875), but the data are not shown and will be presented in future manuscripts.

**Table 2:** Biocidal activities (antibacterial and anticancer) of examined compounds/composites, compared to standard biocidal agents*.

Agents-composites	Antibacterial activity **				Anticancer activity ***	
	*Salmonella typhimurium*		*Staphylococcus aureus*			
	ZOI	MIC	ZOI	MIC	CaCo-2	HeLa
Ch	14.6 ± 2.1^a^	60.0	12.2 ± 1.2^a^	67.5	1423.2 ± 81.5^a^	1852.7 ± 71.6^a^
GCm	16.8 ± 2.4^a^	55.0	15.4 ± 1.6^a^	55.0	528.3 ± 49.3^b^	726.5 ± 51.5^b^
SeNPs	30.2 ± 3.2^b^	35.0	27.5 ± 2.2^b^	35.0	48.9 ± 4.6^c^	69.8 ± 6.1^c^
T1	39.5 ± 5.6^c^	15.0	36.1 ± 4.5^c^	17.5	34.8 ± 4.2^d^	46.9 ± 4.2^d^
T2	34.6 ± 4.1^d^	20.0	29.9 ± 4.2^d^	25.0	25.4 ± 3.1^e^	37.6 ± 2.8^e^
T3	28.3 ± 3.8^e^	27.5	25.6 ± 2.9^e^	32.5	17.7 ± 2.3^f^	29.1 ± 3.3^f^
Ampicillin	28.5 ± 4.2^e^	47.5	25.1 ± 3.5^e^	42.5		
Cisplatin					24.5 ± 2.5^e^	38.3 ± 3.9^e^

* Dissimilar letters (superscript) in one column designate significance at *p* ≤ 0.05. ** ZOI: Mean diameter of inhibition zones in mm ± standard deviation; MIC: minimal inhibitory concentrations (mg/L). *** Expressed as IC_50_ (μg/mL) toward cancerous cell lines, using MTT assay.

Regarding the anticancerous activities of agents/NCs toward CaCo2 and HeLa cells, compared with cisplatin (standard anticancerous chemotherapy), the most effectual anticancer was the T3 composite, as evidenced by the lowest IC_50_ toward both cancerous cells. The T3 anticancer activity significantly exceeded the activity of cisplatin. The HeLa cells were generally more resistant than CaCo2 to the entire inspected agents/NCs.

The bare biopolymers (Ch and GCm) exhibited lower biocidal activities than the NCs toward the examined bacteria and cancerous cells.

The bioactivities of GCm are suggested to correlate with its contents from active phytochemicals; the potential occurrence of phenolic acids in *L. sativum* and GCm includes rosmarinic, carnosic, caffeic, p-coumaric, cinnamic, and ferulic acids, which were emphasized to have potent antibacterial and anticancerous activities (Sharma and Agarwal, 2011; Razmkhah et al., 2016; Haj Bloukh et al., 2021). Furthermore, the combination between *L. sativum* extract and nanometals (e.g., Ag NPs) was reported to reinforce their antibacterial activity (Haj Bloukh et al., 2021), whereas the GCm/AgNPs conjugation was also reported to gain synergistic antibacterial potentialities (Ibrahim et al., 2021), which was validated here with GCm/SeNPs action.

The elevated resistance of G^+ve^ bacteria to microbicidal molecules is suggested to correlate with the thick protective peptidoglycan layers encompassing teichoic/lipoteichoic acids, which provide more barriers for G^+ve^ bacteria to resist nanomaterials penetration into internal cells (Menon et al., 2018; Tayel et al., 2020). Contrarily, G^–ve^ bacterial cells comprise thinner layers of protective peptidoglycan, smaller cross-linkage/condensed membrane, and highly negatively charged lipopolysaccharides in their exterior membranes, which enforce porin channels’ development and enable more penetration of NPs/NCs biocides into inner cells/vital organelles (Reda et al., 2019; Breijyeh et al., 2020; Godlewska-Z Yłkiewicz et al., 2020; Haj Bloukh et al., 2021).

The G^–ve^ bacteria porin channels can selectively permit NPs/NM penetration into cells, correlating with ROS release from SeNPs, which causes the destruction/inactivation of G^–ve^ energetic components (proteins, DNA, enzymes, etc.) (Menon et al., 2018; Gad et al., 2021).

The synergistic bioactivities in conjugated NCs formulation (e.g., T1, T2, and T3), compared with their plain constituents (Ch, GCm, and SeNPs) were attributed earlier to NCT capability (with its high positive charges) to uphold other bioactive nanomaterials, affix the negative cells/membranes, and disrupt their permeability and potentialities to inhibit bacterial biosystems (Wazed et al., 2010; Al-Saggaf et al., 2020; Tayel et al., 2021). The powerful antibacterial potentiality of (T1) NC could be mostly due to its surface positivity, which facilitates its particles’ attachment with negatively charged bacterial walls/membranes and vital components (Larsson et al., 2012). The biosynthesized nanometals (e.g., SeNPs) were established to own powerful bactericidal actions, depending chiefly on ROS generation and cytotoxicity toward challenging cells via interaction/inactivation of their metabolic pathways (Breijyeh et al., 2020; Długosz et al., 2020; ElSaied et al., 2021). The conjugation of such nanometals with coating biopolymers could impressively diminish their potential toxicity toward mammals’ tissues/cells but favorably maintain or somewhat increase their bioactivities against microbes and cancerous cells (Saravanakumar et al., 2012; Długosz et al., 2020; Zienkiewicz-Strzałka and Deryło-Marczewska, 2020). Although the antibacterial assay of NCs is involved in qualitative/quantitative assays, the use of more assays (e.g., Time-kill kinetics assays) is suggested for more understanding of the interactions between the microbial cells and NCs.

### 3.4. Assessment of nanocomposites’ antibacterial action via SEM

**Figure 3:**
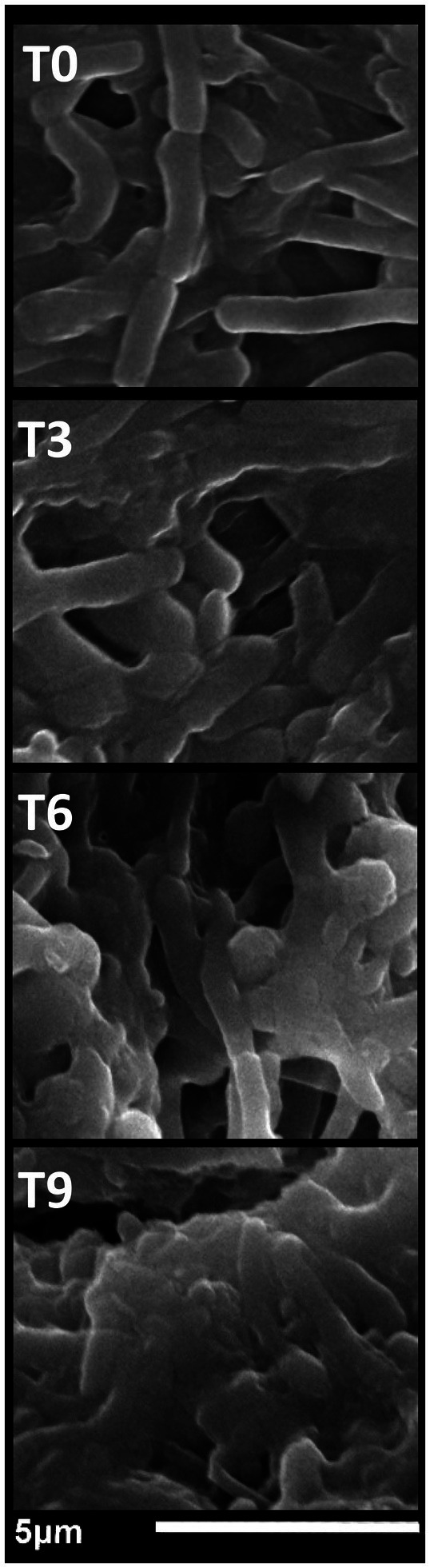
The antibacterial impact of chitosan/garden cress/SeNPs nanocomposites on the morphology of *Salmonella typhimurium* after exposure for 0 h (T0), 3 h (T3), 6 h (T6), and 9 h (T9).

For an imaginable explanation of NCT/GCm/SeNPs (formulation T1) antibacterial actions, SEM visualizations were screened for exposed *S. typhimurium* cells (with the utmost sensitivity) to NCs after 3 h, 6 h, and 9 h compared with 0-time exposure (Figure 3). The cells in the 0-time treatment had wholesome, ordinary, and smooth structures, with the least signs of deformations or distortions (Figure 3-T0). After NCs exposure for 3 h, notable deformation/distortion indications were observable on bacterial surfaces, with some appearing NCs particles attached to bacteria (Figure 3-T3). The deformations, distortions, and lysis of *S. typhimurium* cells became more observable after exposure to NC for 6 h (Figure 3-T6); the NCs of NCT/GCm/SeNPs were very observable in combination with lysed cells’ residues, and the cells mostly mislaid their contacted/uniformed membranes. After 9 h of NCs exposure, almost all bacterial cells were decomposed and lysed; their liberated interior constituent and wall residues were the only observable things, in combination with the particles of NCT/GCm/SeNPs (Figure 3-T9). Harmonized observations were evident after the treatments of diverse bacterial species with NCT-based NCs that enclosed NM or phytocompounds (Elnagar et al., 2021; Hashem et al., 2022; Youssef et al., 2022).

The NCT role in the NC antibacterial actions was distinct from the captured images, principally because of NCT’s competency to adhere onto microbes, with the added bactericides, and expedite their actions after penetrating cells (Manivasagan and Oh, 2016; Al-Saggaf et al., 2020; Tayel et al., 2021; Shehabeldine et al., 2022).

From the captured SEM images of treated *S. typhimurium* with NCs, various potential antibacterial mechanisms could be suggested, originating from synergism among reacting nanomolecules and include an attachment to cell walls/membranes, suppression of cell walls synthesis, penetration into the innermost cells, boosted membranes’ permeability, leakages of fundamental components, the suppression of metabolic bioactivities/pathways, and interaction with cells’ crucial constituents (Menon et al., 2018; Gad et al., 2021).

### 3.5. Assessment of nanocomposite anticancer action via fluorescent imaging

**Figure 4:**
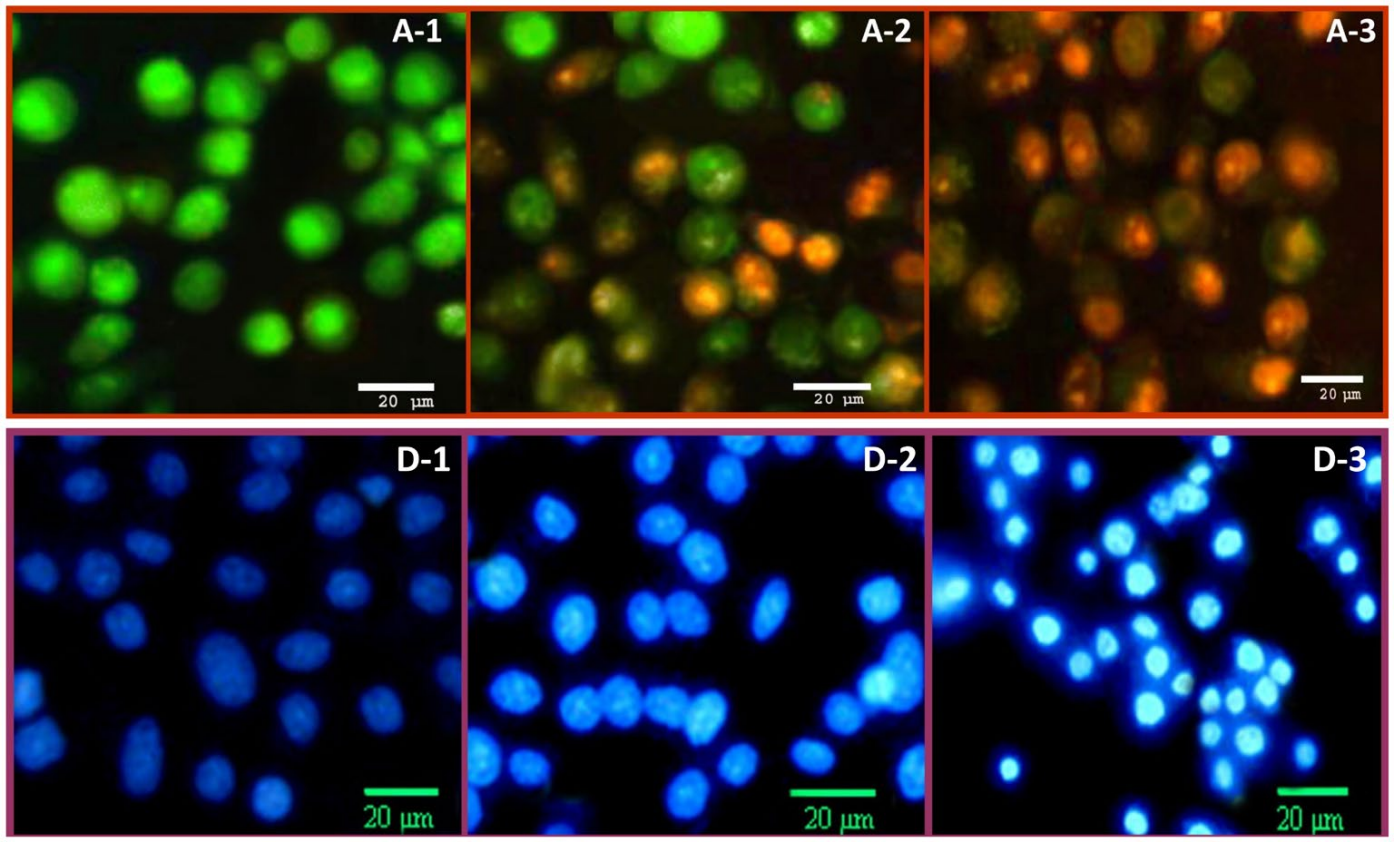
The anticancer impact of chitosan/garden cress/SeNPs nanocomposites on the apoptosis of HeLa cells, stained with (A): dual acridine orange/ethidium bromide and (D): DAPI staining after treatment for 24 h (A2 and D2) and 48 h (A3 and D3), compared to control cells (A1 and D1).

The potential apoptosis/necrosis signs of HeLa cells, after treatment with NCT/GCm/SeNPs (formulation T3), were additionally elucidated via double AO/EB fluorescent staining (Figure 4, A-1,2,3) and DAPI staining (Figure 4, D-1,2,3). In the AO/EB assessment, the control cancerous cells had uniformed and intact assemblies, with abundant green staining for both cells and their nuclei (Figure 4, A-1). After 24 h of exposure (Figure 4, A-2), early apoptosis marks had emerged including notable chromatin condensation and remarkable nuclei with bright green staining; many exposed cells’ color turned orange (mixed with green) at this stage. The late apoptosis/ secondary necrosis signs were evidently visualized in treated HeLa cells for 48 h (Figure 4, A-3), which included more condensation of chromatins, the abundance of stained dense orange parts, and the vanishing of the most greenish stained cells’ parts.

The DAPI staining of HeLa cells treated with NCT/GCm/SeNPs (formulation T3) indicated further apoptosis signs in cancerous cells (Figure 4, D). The control cells (Figure 4, D-1) appeared with a bale blue stain, uniformed structures, and a healthy appearance. The apoptosis signs were evidently visualized after 24 h of NC treatment (Figure 4, D-2), including the bright blue staining of most cells and nuclei. The anticancerous action of NCT/GCm/SeNPs could be claimed to have time-depending attributes; after a 48 h exposure (Figure 4, D-3), most cancerous cells had obvious and vigorous late apoptosis indicators that included cells’ shrinkage and rounding, membranes’ blebbing, and more bright blue staining for the entire exposed cells.

Formulation T3 (with NCT:GCm/SeNPs ratio of 1:2; zeta potential = −25.8 mV) was the most effectual as an anticancer treatment for killing cancerous cells, which indicates the implication of NPs charge in anticancer action (Larsson et al., 2012). Both GCm and SeNPs were negatively charged and the net charge of T3 was negative also (Table 1), whereas the NCT was positively charged, which indicates that negative molecules are more effective for combating cancer cells (Larsson et al., 2012; Alalawy et al., 2020; Ibrahim et al., 2021). The GCm anticancer activity was reported, either alone or conjugated with AgNPs, against cancerous colon cells, which exhibited notable markers of apoptosis and mitotic arresting (Ibrahim et al., 2021). The GCm was biosafe for normal cells and even helpful for their deviation, whereas the bioactive molecules of GCm (e.g., flavonoids and isothiocyanates) and the generation of ROS were the potential factors for tumor cells’ apoptosis induction, p53 gene expression increase, and cell division arrest (Alqahtani et al., 2019; Zhao et al., 2020; Ibrahim et al., 2021).

The actions of biosynthesized SeNPs as anticancerous agents were also reported recurrently; numerous tumor cells were affected by the biogenic SeNPs treatments (Vahidi et al., 2020; Spyridopoulou et al., 2021; Ullah et al., 2022). The biogenic SeNPs were able to induce pro-apoptotic, cytotoxicity, and immunogenic cell deaths via different suggested mechanisms including the activation of apoptotic pathways, ROS generation, cell cycle arresting, cell homeostasis disruption, DNA fragmentation, and mitochondrial dysfunction (Vahidi et al., 2020; Spyridopoulou et al., 2021; Ullah et al., 2022). Unlike their actions toward bacterial cells, the conjugation of SeNPs with NCT or other encapsulated nanopolymers could synergize their activities toward cancerous cells, while diminishing their biotoxicity for somatic normal cells (Saravanakumar et al., 2012; Długosz et al., 2020; Ullah et al., 2022).

## 4. Conclusion

To achieve innovative bioactive NCs with higher efficacy and lower biotoxicity, inventive NCs were formulated from NCT, GCm, and GCm-mediated SeNPs for potential applications as antibacterial and anticancer agents. The synthesis of SeNPs (with 12.7 nm mean particles’ size) was achieved using GCm, and different formulations of NCs from NCT {GCm/SeNPs [T1 (2:1); T2 (1:1); and T3 (1:2)]} were constructed with mean particles’ sizes of 316.4, 252.8, and 127.3 n, respectively. The inventive NCs exhibited potent bioactivities against G^−^ (*S. typhimurium*), G^+^ (*S. aureus*) bacteria, and cancerous cells (CaCo-2 and HeLa), which exceeded the efficacy of standard biocides (the antibacterial Ampicillin and the anticancer Cisplatin). Formulation T1 (2 NCT: 1 GCm/SeNPs) was the most effectual as an antibacterial NC, whereas formulation T3 (1 NCT: 2 GCm/SeNPs) was the superior NC as an anticancer. The innovative construction and biological nature of NCs components, with the augmented biosafety of employed biopolymers, could strongly enhance their prospective applications for controlling pathogenic bacteria and fighting cancerous cells. Based on their promising antibacterial and anticancerous potentialities, the practical employments of inventive NCT/GCm/SeNPs NCs, as food preservatives and dietary anticancer treatments, are highly advised to be investigated in prospective works.

## Data availability statement

The datasets presented in this study can be found in online repositories. The names of the repository/repositories and accession number(s) can be found in the article/supplementary material.

## Author contributions

ME-S, MO, ME-H, SA, SA-G, RE, HM, and AT contributed to the conception, design, investigation, and validation of the study and performed the statistical analysis and drafted the manuscript. MME, MO, ME-H, SA, and SA-G contributed to resources/funding acquisition. HM and AT supervised the work. All authors contributed to the article and approved the submitted version.

## Funding

The work was funded by Ministry of Education and King Abdulaziz University, Jeddah, Saudi Arabia under the grant no (IFPRC-140-150-2020).

## Conflict of interest

The authors declare that the research was conducted in the absence of any commercial or financial relationships that could be construed as a potential conflict of interest.

## Publisher’s note

All claims expressed in this article are solely those of the authors and do not necessarily represent those of their affiliated organizations, or those of the publisher, the editors and the reviewers. Any product that may be evaluated in this article, or claim that may be made by its manufacturer, is not guaranteed or endorsed by the publisher.
