# Corrigendum: Fabrication of bioactive nanocomposites from chitosan, cress mucilage, and selenium nanoparticles with powerful antibacterial and anticancerous actions

**DOI:** 10.3389/fmicb.2025.1579721

**Published:** 2025-03-28

**Authors:** Mohsen M. El-Sherbiny, Mohamed I. Orif, Mohamed E. El-Hefnawy, Sultan Alhayyani, Soha T. Al-Goul, Rawan S. Elekhtiar, Hoda Mahrous, Ahmed A. Tayel

**Affiliations:** ^1^Department of Marine Biology, Faculty of Marine Sciences, King Abdulaziz University, Jeddah, Saudi Arabia; ^2^Department of Marine Chemistry, Faculty of Marine Sciences, King Abdulaziz University, Jeddah, Saudi Arabia; ^3^Department of Chemistry, College of Sciences and Arts, King Abdulaziz University, Rabigh, Saudi Arabia; ^4^Department of Fish Processing and Biotechnology, Faculty of Aquatic and Fisheries Sciences, Kafrelsheikh University, Kafr el-Sheikh, Egypt; ^5^Genetic Engineering and Biotechnology Research Institute, University of Sadat City, Sadat, Egypt

**Keywords:** anticancer, antimicrobial, biocidal activities, biopolymers nanocomposites, biosynthesis

In the published article, there was an error in Figure 3 page 8 as published. There was a mistake in Figure 3 that was incorrectly uploaded to submission system in original manuscript. The correct figure was provided in the corrected version. The corrected Figure 3 and its caption appear below.

**Figure 3:**
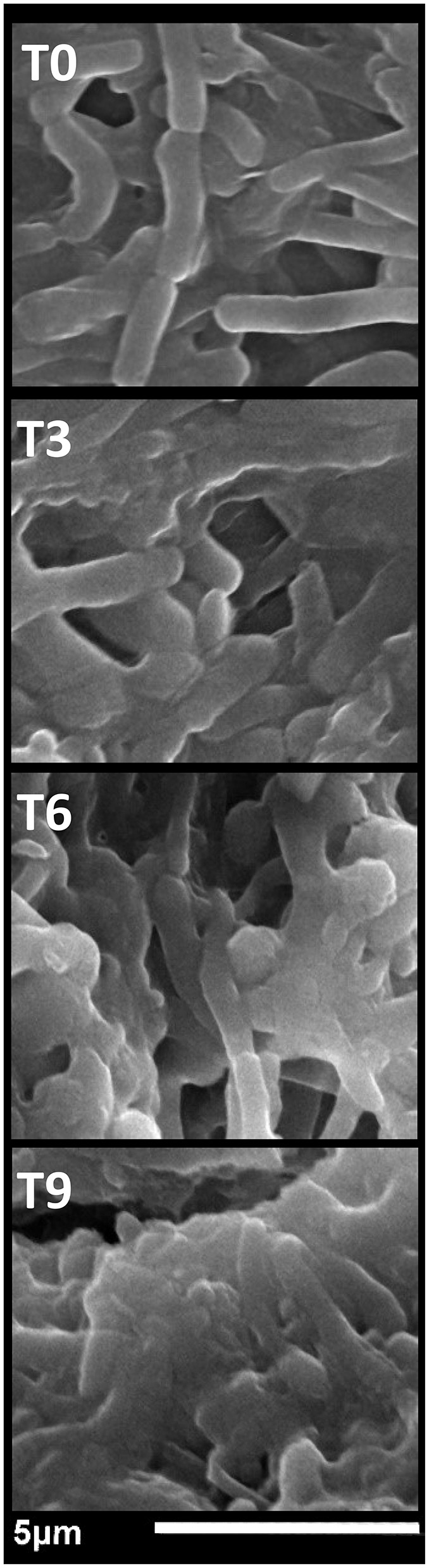
The antibacterial impact of chitosan/garden cress/SeNPs nanocomposites on the morphology of *Salmonella typhimurium* after exposure for 0 h (T0), 3 h (T3), 6 h (T6), and 9 h (T9).

The authors apologize for this error and state that this does not change the scientific conclusions of the article in any way. The original article has been updated.

