# Supplementary material for: Fabrication of bioactive nanocomposites from chitosan, cress mucilage, and selenium nanoparticles with powerful antibacterial and anticancerous actions
Source: Front Microbiol. 2023 Jul 21;14:1210780. doi: 10.3389/fmicb.2023.1210780 (PMC10402636; doi:10.3389/fmicb.2023.1210780)
Supplement: Supplementary file 1 [file Data_Sheet_1.PDF]

# Supplementary Materials

## Fabrication of bioactive nanocomposites from chitosan, cress mucilage and selenium nanoparticles with powerful antibacterial and anticancerous actions

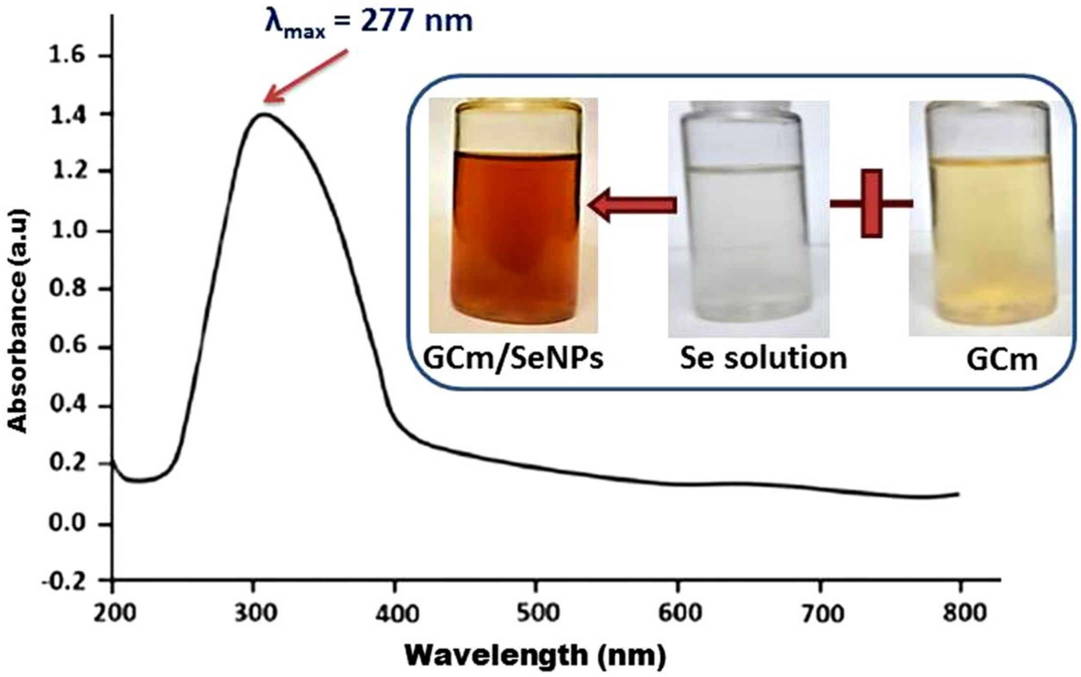


**Fig 1-S: Visual color change (right photos) after Se reduction with garden cress mucilage (GCm), and the UV-vis spectrum of biosynthesized GCm/SeNPs (left curve).**

The UV-Vis spectrum of GCm/SeNPs solution was documented spectrophotometerically (UV-2450, Shimadzu, Japan), within 200‑800 nm absorbance range. The figure appointed the color change to deep orange after reduction of SeNPs with GCm; the maximum absorbance of GCm/SeNPs was recorded at 277 nm, which agree with former reports investigated SeNPs biosynthesis.

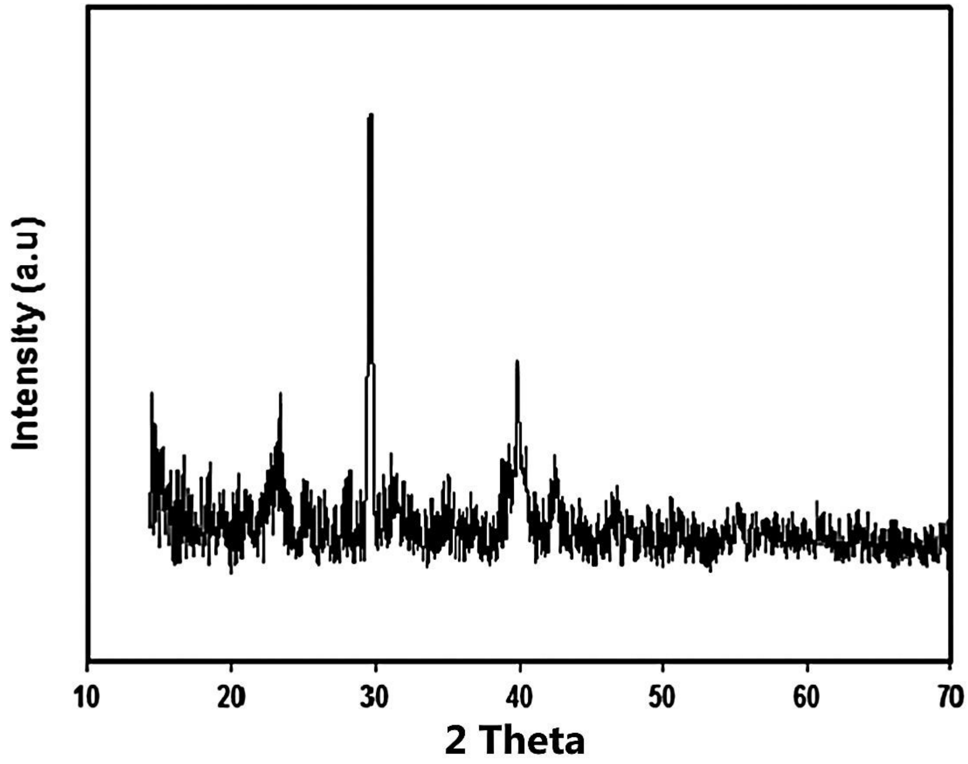


**Fig 2-S: X-ray Diffraction (XRD) pattern of biosynthesized SeNPs with garden cress mucilage**

The X-ray Diffraction (XRD) pattern of GCm/SeNPs was measured using diffractometer (XRD-6000, Shimadzu, Japan) with Cu-kα radiation (λ = 1.5414 Å) at 30 mA and 40 KV within 10‑70° 2θ range. The XRD pattern of GCm-mediated SeNPs revealed the formation of crystalline NPs with notable noise arising from their biosynthesis. The main diffraction peaks were detected at 23.58° (100), 29.63° (101), 41.02° (110), 43.64° (102), and 46.48° (111), respectively, which validated the effectual synthesis of SeNPs.

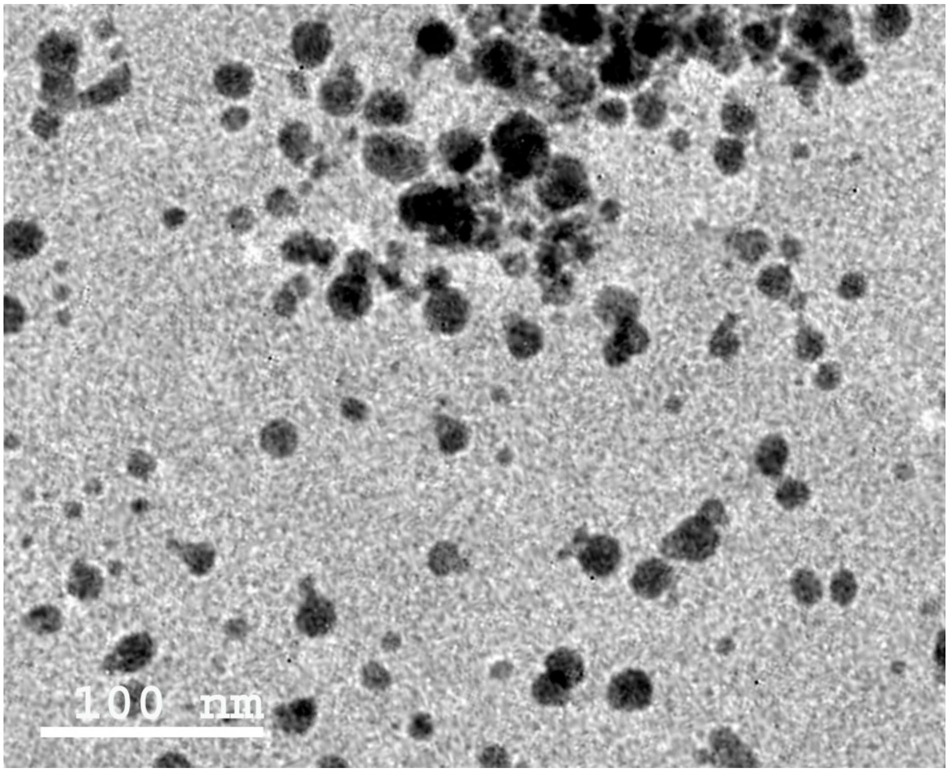


**Fig 3-S: Transmission electron (TEM) microscopic image of biosynthesized SeNPs with garden cress mucilage**

The Transmission electron microscope “TEM, JEM-100CX, JEOL” was operated for screening the apparent shape, size and distributions of GCm/SeNPs. The TEM imaging of GCm-mediated SeNPs revealed their effectual synthesis, spherical shapes, homogenous sizes and distributions. The average particles’ size was 12.83 nm, which harmonized the illustrated data of DLS analysis.

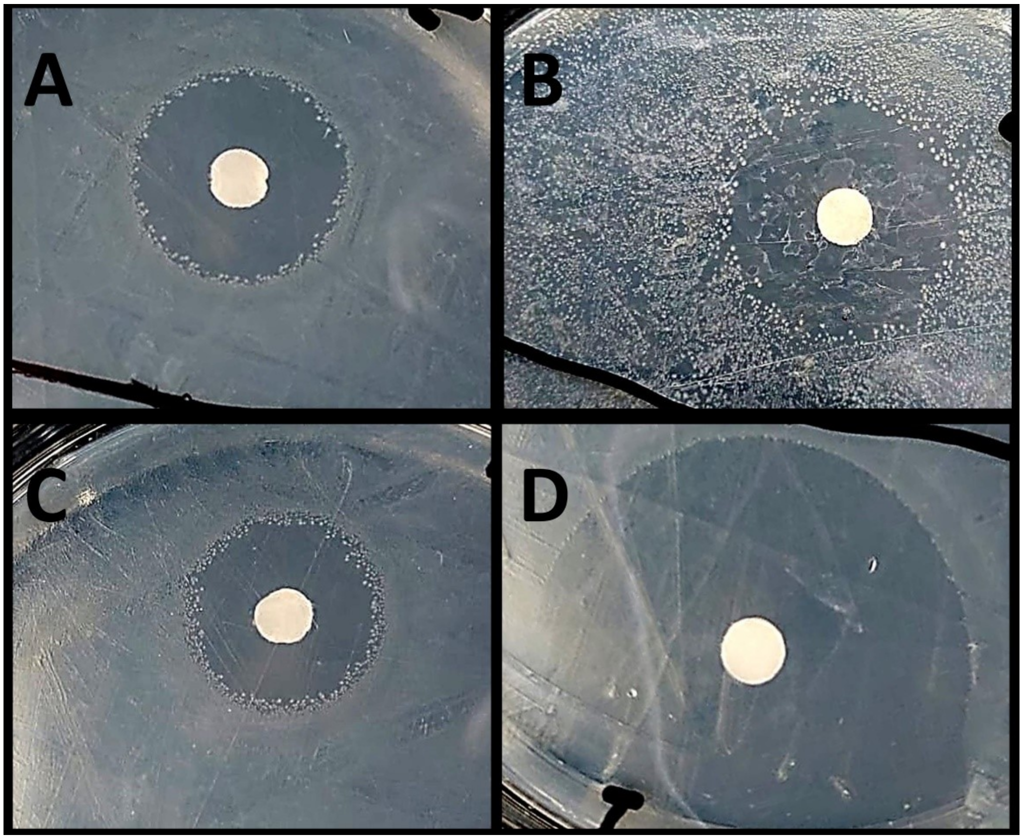


**Fig 4-S: Examples of appeared growth inhibition after challenging bacterial pathogens with fabricated nanocomposites**

A: *S. typhimurium* challenging with T3 composite (1:2; NCT:GCm/SeNPs)

B: *S. aureus* challenging with T2 composite (1:1; NCT:GCm/SeNPs)

C: *S. aureus* challenging with T3 composite (1:2; NCT:GCm/SeNPs)

D: *S. typhimurium* challenging with T1 composite (2:1; NCT:GCm/SeNPs)
